# Association between dietary minerals and glioma: A case-control study based on Chinese population

**DOI:** 10.3389/fnut.2023.1118997

**Published:** 2023-03-02

**Authors:** Weichunbai Zhang, Yongqi He, Xun Kang, Ce Wang, Feng Chen, Zhuang Kang, Shoubo Yang, Rong Zhang, Yichen Peng, Wenbin Li

**Affiliations:** Department of Neuro-Oncology, Cancer Center, Beijing Tiantan Hospital, Capital Medical University, Beijing, China

**Keywords:** minerals, glioma, case-control study, Chinese population, dose-response relationship

## Abstract

**Background:**

As one of the essential nutrients for the human body, minerals participate in various physiological activities of the body and are closely related to many cancers. However, the population study on glioma is not sufficient.

**Objective:**

The purpose of this study was to evaluate the relationship between five dietary minerals and glioma.

**Methods:**

A total of 506 adult patients with glioma and 506 healthy controls were matched 1:1 according to age (±5 years) and sex. The food intake of the subjects in the past year was collected through the food frequency questionnaire, and the intakes of calcium, magnesium, iron, zinc, and copper in the diet were calculated. The logistic regression model was used to estimate the odds ratio (OR) and 95% confidence interval (95% CI) for dietary minerals to gliomas.

**Results:**

After adjusting for confounders, higher intakes of calcium (OR = 0.65, 95% CI: 0.57–0.74), magnesium (OR = 0.18, 95% CI: 0.11–0.29), iron (OR = 0.04, 95% CI: 0.02–0.11), zinc (OR = 0.62, 95% CI: 0.54–0.73), and copper (OR = 0.22, 95% CI: 0.13–0.39) were associated with a significantly decreased risk of glioma. Similar results were observed in gliomas of different pathological types and pathological grades. The restriction cubic spline function suggested significant linear dose-response relationships between intakes of five minerals and the risk of glioma. When the dietary minerals exceeded a particular intake, the risk of glioma stabilized.

**Conclusion:**

Our study suggests that higher dietary intakes of calcium, magnesium, iron, zinc, and copper are associated with a decreased risk of glioma. However, the results of this study require further exploration of potential mechanisms in the future better to elucidate the effects of mineral intake on gliomas.

## Introduction

Gliomas are the most common type of primary central nervous system tumor, accounting for about 80.7% of malignant brain tumors (1). Although the incidence of glioma is very low, only 5.47/100,000 (1), due to the high mortality rate of glioma and the large population base in China, it caused serious disease burden and economic burden to families. Therefore, exploring modifiable factors in the etiology of glioma was essential to provide substantial scientific support for primary prevention.

Compared with other cancers, the etiology of gliomas was largely uncertain and complex. Although gliomas have long been reported to be related to head trauma (2), allergies (3), use of mobile phones (4), and occupational exposure (5). Ionizing radiation was still the only apparent environmental risk factor (6). In recent years, the relationship between diet and glioma has attracted more and more attention (7). Existing studies have found that dietary patterns (8), food groups (9), and nutrients (10) all had certain effects against gliomas. In particular, the vitamins and phytochemicals in these foods had certain antioxidant effects, protecting healthy tissues from oxidative stress-induced damage and inhibiting the occurrence and development of glioma (7, 11–13). However, previous studies on diet and glioma ignored minerals with similar effects. These elements also played an antioxidant (14), anti-inflammatory (15), and anti-tumor (16) effect on the body. Therefore, several common minerals, such as calcium (Ca), iron (Fe), and zinc (Zn), may also affect gliomas. Yekta et al. found a significant negative association between dietary Ca intake and glioma (OR = 0.23, 95% CI: 0.08–0.65) in an Iranian hospital-based case-control study (17). This association was also found in the San Francisco Bay Area Adult Glioma Study but was only significant in women (18). Chen et al. also found that the consumption of intracellular Ca ions affected the abnormal growth of C6 glioma cells by affecting the signal transduction of the endoplasmic reticulum (19). Studies on Fe and gliomas had similar findings. Ward et al. followed up for 14.1 years in a European prospective cohort study to explore the effect of meat and heme Fe on gliomas but found no association between them [Hazard ratio (HR) = 0.96, 95% CI: 0.73–1.26] (20). However, *in vivo* exposure, a higher concentration of toenail Fe was found to have a protective effect against gliomas (OR = 0.42, 95% CI: 0.19–0.95) (21). Although cell experiments found that low Zn can inhibit the cell growth of rat glioma C6 cells (22), Dimitropoulou et al. did not find any significant association between dietary Zn and glioma in the nutritional epidemiological study (23). The influence of magnesium (Mg) and copper (Cu) on glioma was far less than that of other elements, and their related studies mainly focused on metalloproteins. Bioinformatics studies have found differential expressions of various copper-related proteins in gliomas and normal tissues (24). The overexpression of Mg transporter 1 was also associated with the occurrence and progression of gliomas (25).

Although experimental data on the role of minerals in the prevention of glioma were promising, the vast majority of studies have focused on *in vitro* assays. Epidemiological studies on minerals and gliomas were insufficient. On the one hand, no studies have reported their dose-response relationship. On the other hand, relevant studies were mainly based on the population of European and American populations, with geographical limitations. Therefore, to further explore the association between dietary minerals and glioma, we investigated the association of five typical dietary mineral intakes with the risk of glioma in the case-control study based on a Chinese population and attempted to delineate the dose-response relationship between the two aim to provide some epidemiological evidence for the prevention of glioma by five minerals.

## Methods

### Study population

One thousand and twelve subjects (506 cases and 506 controls) participated in the diet and glioma case-control study at Beijing Tiantan Hospital, Capital Medical University, between 2021 and 2022. The case group consisted of patients with glioma who were recently diagnosed by neuro-oncologists and pathologists according to the 2021 neuro-tumor diagnostic criteria (26). Among them, there were 104 cases of astrocytoma, 67 cases of oligodendroglioma, 237 cases of glioblastoma, and 98 cases of other gliomas (including 18 cases of diffuse midline glioma). The control groups were recruited from healthy individuals in the community and matched 1:1 with cases by age and sex. All participants were ≥18 years of age. Among them, 15 people in the case group refused to participate, with a response rate of 97.2%, and 41 people in the control group refused to participate, with a response rate of 92.7% (Supplementary Figure 1). On this basis, they were excluded according to certain conditions, including suffering from digestive, neurological, and endocrine system diseases, suffering from other cancers, significant dietary behavior changes such as dieting before the investigation, abnormal energy intake (>5,000 or <400 kcal/d), pregnant women and nursing mothers, and taking drugs such as hormones. All participants provided informed consent, and the study protocol was approved by the Institutional Review Board of Beijing Tiantan Hospital, Capital Medical University (No.KY2022-203-02).

### Data collection

The required information was obtained through questionnaires. Through face-to-face interviews, investigators collected data on demographics, lifestyle habits, disease history, and dietary intake and measured some anthropometric indicators. Demographic data included date of birth, sex, occupation, education level, and household income. Lifestyle habits included living conditions in high-risk areas, smoking status, drinking status, and physical activity. Living near electromagnetic fields and broadcast antennas in the past 10 years have been defined as high-risk residential areas (4). Physical activity was assessed using the International Physical Activity Questionnaire (27). Disease histories were collected for diseases potentially associated with glioma, including allergies, head trauma, and other cancers.

Anthropometric data mainly consisted of height and weight, which were collected by trained staff using standardized techniques and calibrated equipment. Body mass index (BMI) was calculated by dividing weight (in kilograms) by the square of height (in meters), and the result was accurate to two decimal places.

Dietary intakes were assessed through the 114-item food frequency questionnaire (Supplementary Table 1). The questionnaire has been validated in previous studies (28). According to the foods reported in the literature that may affect the risk of glioma, several foods were added and deleted on this basis to make the food frequency questionnaire more suitable for the research needs. To improve the accuracy of the dietary survey, the investigators collected the dietary intake information of the study subjects in the past year through face-to-face interviews by providing pictures of different food volumes and qualities. The study subjects need to fill in the intake of each food according to their conditions, including whether the food was consumed, the frequency of intake (number of intakes per day/week/month), and the average intake per time. In order to further verify the reproducibility and validity of the questionnaire in this study, after about 1 year, we investigated 30 healthy controls again, collected the dietary information of the subjects through the food frequency questionnaire and 24-h recall (two working days and one rest day), calculated the food consumption and nutrient intake, and evaluated the reproducibility and validity of the questionnaire by the mean and correlation coefficient. For reproducibility, the correlation coefficients of food group were 0.502–0.847, and that of nutrients were 0.437–0.807. For validity, the correlation coefficients of food group were 0.381–0.779, and the correlation coefficients of nutrients were 0.380–0.804 (Supplementary Tables 2–5).

The study involved five common minerals, including Ca, Mg, Fe, Zn, and Cu. The intakes of all minerals were calculated based on the information of each food item and the Chinese Food Composition Table (29). The daily intakes of various foods were calculated according to the frequency of food intake and the amount of each intake filled in by the study subjects. The “Chinese Food Composition Table” provided the content of five minerals per unit of food. It was calculated by multiplying the daily intake of various foods and the unit content of minerals in the food. Then the sum of the intakes of all minerals in different foods was calculated as the total intake. Energy intake polyunsaturated fatty acids (PUFAs) were calculated similarly.

### Statistical analysis

Demographics, lifestyle habits, and disease history were characterized using descriptive analysis. The *t*-test was used for normally distributed continuous variables, and the chi-square test was used for categorical variables to compare general characteristics between pathological subtypes and controls. The Mann-Whitney *U*-test was used to compare mineral intakes between case and control groups, and Spearman's correlation coefficient was used to evaluate the correlation between the five minerals. We used logistic regression models to estimate ORs and 95% CI between mineral intake and the risk of glioma, adjusting for potential confounders. In this analysis, each mineral intake was divided into tertiles, with the lowest tertile as the reference group. In addition, mineral intake was also brought into the model as a continuous variable.

Potential confounding variables included age, BMI, occupation (manual workers, mental workers, or others), educational levels (primary school and below, middle school, or university and above), household income (below 3,000 ¥/month, 3,000–10,000 ¥/month, or above 10,000 ¥/month), high-risk residential areas (yes or no), smoking status (never smoking, former smoking, or current smoking), drinking status (non-drinker, occasional drinker, or frequent drinker), history of allergies (yes or no), history of head trauma (yes or no), family history of cancer (yes or no), physical activity (low, moderate, or violent), PUFAs intake, and energy intake.

Age, sex, BMI, occupation, education level, household income, smoking status, history of allergies, family history of cancer, and physical activity were also used as the basis for subgrouping, and subgroup analyses were performed by logistic regression after adjusting for confounding factors. In addition, to overcome the inherent limitations of elemental analysis as a grade variable, the dose-response relationship was analyzed using the restricted cubic spline function in the logistic regression model after adjusting for confounders, with nodes distributed in the 20th, 40th, 60th, and 80th percentiles, the reference value (OR = 1) was set at the 10th percentile (30).

All statistical analyses were performed using SPSS 26.0 and R 4.1.1. A two-sided *P*-value < 0.05 was used to determine the statistical significance.

## Results

### Study population and mineral characteristics

The patients with glioma of different pathological subtypes and their corresponding control groups were completely identical in sex composition and similar in age distribution. Compared with controls, glioma patients differed in BMI (*P* < 0.001), occupation (*P* = 0.024), education levels (*P* < 0.001), household income (*P* < 0.001), smoking status (*P* = 0.039), drinking status (*P* < 0.001), physical activity (*P* < 0.001), history of allergies (*P* < 0.001), and family history of cancer (*P* = 0.001). Patients with various pathological subtypes of glioma had higher BMI, slightly lower education levels, lower household income, more drinkers, and more physical activity, which was consistent with the overall population. In addition, only the population with glioblastoma had a higher family history of cancer (*P* = 0.006). Other glioma populations had more manual workers (*P* = 0.002) and a lower history of allergies (*P* = 0.004). In other respects, there were no significant differences (Table 1).

**Table 1:** Basic characteristics of the study participants.

	**Astrocytoma**			**Oligodendroglioma**			**Glioblastoma**			**Others**			***P* ^b^**
	**Case**	**Control**	***P** ^a^*	**Case**	**Control**	***P** ^a^*	**Case**	**Control**	***P** ^a^*	**Case**	**Control**	***P** ^a^*	
Age (years)	39.32 ± 13.43	38.01 ± 13.01	0.477	39.52 ± 9.75	37.66 ± 9.67	0.268	45.27 ± 13.07	43.75 ± 12.89	0.202	41.83 ± 13.58	40.58 ± 13.27	0.517	0.072
Sex (%)			1.000			1.000			1.000			1.000	1.000
Male	58.7	58.7		61.2	61.2		55.3	55.3		52.0	52.0		
Female	41.3	41.3		38.8	38.8		44.7	44.7		48.0	48.0		
BMI	24.02 ± 3.05	22.89 ± 3.34	0.012	24.37 ± 2.91	23.27 ± 3.64	0.056	23.99 ± 3.30	23.13 ± 3.12	0.004	23.90 ± 3.56	22.89 ± 3.33	0.042	<0.001
High-risk residential area (%)			0.222			0.662			0.729			0.319	0.534
Yes	23.1	16.3		17.9	20.9		19.0	20.3		27.6	21.4		
No	76.9	83.7		82.1	79.1		81.0	79.7		72.4	78.6		
Occupation (%)			0.406			0.931			0.119			0.002	0.024
Manual workers	23.1	17.3		32.8	31.4		22.4	21.1		35.7	14.3		
Mental workers	61.5	61.5		55.2	58.2		51.0	59.5		43.9	63.3		
Others	15.4	21.2		12.0	10.4		26.6	19.4		20.4	22.4		
Education level (%)			0.013			0.009			<0.001			<0.001	<0.001
Primary school and below	2.9	4.8		6.0	0		6.3	2.5		1.3	2.0		
Middle school	43.3	24.0		40.3	23.9		41.8	26.2		39.8	24.5		
University and above	53.8	71.2		53.7	76.1		51.9	71.3		46.9	73.5		
Household income (%)			0.007			<0.001			<0.001			0.024	<0.001
<3,000 ¥/month	11.5	18.3		10.4	19.4		7.2	19.8		13.3	13.3		
3000–10,000 ¥/month	72.1	51.0		83.6	47.8		77.6	46.8		70.4	54.0		
>10,000 ¥/month	16.3	30.8		6.0	32.8		15.2	33.4		16.3	32.7		
Smoking status (%)			0.615			0.405			0.382			0.079	0.039
Never smoking	70.2	73.1		64.2	74.6		73.0	74.7		66.3	79.6		
Former smoking	10.6	6.7		16.4	10.4		13.1	9.3		12.3	5.1		
Current smoking	19.2	20.2		19.4	15.0		13.9	16.0		21.4	15.3		
Drinking status (%)			0.010			0.003			<0.001			0.003	<0.001
Non-drinker	61.5	58.7		59.7	58.3		64.6	55.3		68.4	55.1		
Occasional drinker	10.6	25.0		11.9	31.3		13.9	30.8		13.2	33.7		
Frequent drinker	27.9	16.3		28.4	10.4		21.5	13.9		18.4	11.2		
History of allergies (%)			0.122			0.171			0.167			0.004	<0.001
Yes	7.7	14.4		7.5	14.9		8.0	11.8		7.1	21.4		
No	92.3	85.6		92.5	85.1		92.0	88.2		92.9	78.6		
History of head trauma (%)			0.675			0.069			0.451			0.345	0.474
Yes	13.5	11.5		13.4	4.5		9.3	11.4		12.2	8.2		
No	86.5	88.5		86.6	95.5		90.7	88.6		87.8	91.8		
Family history of cancer (%)			0.080			0.395			0.006			0.616	0.001
Yes	30.8	20.2		23.9	17.9		33.3	21.9		25.5	22.4		
No	69.2	79.8		76.1	82.1		66.7	78.1		74.5	77.6		
Physical activity, (%)			0.003			<0.001			<0.001			<0.001	<0.001
Low	16.3	35.6		13.4	50.7		14.3	47.7		9.2	49.0		
Moderate	40.4	37.5		44.8	40.3		41.8	35.4		38.8	34.7		
Violent	43.3	26.9		41.8	9.0		43.9	16.9		52.0	16.3		

^a^*P*-values were derived from Student's *t*-tests for continuous variables according to the data distribution and the chi-square test for the classified variables.

^b^Results of the overall case group and the overall control group.

In terms of dietary intakes, compared with controls, cases had higher intakes of refined grains (*P* < 0.001) and alcohol (*P* < 0.001), and lower intakes of whole grains (*P* < 0.001), legume and products (*P* < 0.001), tubers (*P* < 0.001), vegetables (*P* < 0.001), fungi and algae (*P* < 0.001), fruits (*P* < 0.001), fish and seafood (*P* < 0.001), and dairy products (*P* = 0.025). For other food groups, there was no significant difference (Supplementary Table 6).

In terms of mineral intakes, as shown in Table 2, the intakes of Ca, Mg, Fe, Zn, and Cu in the control group were all significantly higher than those in the case group. In addition, there were significant correlations between individual mineral intakes (Spearman coefficients ranged from 0.709 to 0.919) (Supplementary Table 7).

**Table 2:** Dietary minerals intakes of study participants.

**Minerals**		**Q1**	**Q2**	**Q3**	**Q4**	***P*-value**
Ca (mg/d)	Case	233.07 ± 61.02	411.04 ± 52.37	573.68 ± 51.32	941.45 ± 290.89	<0.001
	Control	203.90 ± 79.60	406.71 ± 51.36	592.04 ± 55.34	1,028.21 ± 358.82	
Mg (mg/d)	Case	161.51 ± 35.32	241.55 ± 19.45	318.91 ± 31.33	502.50 ± 140.23	<0.001
	Control	145.72 ± 43.22	241.06 ± 20.13	331.08 ± 31.10	529.23 ± 135.73	
Fe (mg/d)	Case	9.30 ± 1.81	13.29 ± 1.11	17.27 ± 1.37	27.01 ± 8.74	<0.001
	Control	8.24 ± 2.45	13.11 ± 1.04	17.40 ± 1.35	27.00 ± 7.37	
Zn (mg/d)	Case	5.09 ± 1.09	7.39 ± 0.65	9.79 ± 0.68	14.58 ± 4.23	0.002
	Control	4.50 ± 1.33	7.54 ± 0.66	9.84 ± 0.72	15.07 ± 3.83	
Cu (mg/d)	Case	0.76 ± 0.17	1.14 ± 0.11	1.57 ± 0.17	2.77 ± 0.91	<0.001
	Control	0.66 ± 0.21	1.15 ± 0.11	1.62 ± 0.15	2.80 ± 0.87	

Q1, Q2, Q3, and Q4 represented the quartiles of mineral intake.

### Association between dietary minerals and glioma

The results of the association between minerals with glioma are shown in Table 3. After adjustment for confounding variables (Model 2), the results for the mineral categorical variable showed that individuals with the highest Ca intake was associated with a 90% decreased risk of glioma compared with the first tertile (OR = 0.11, 95% CI: 0.05–0.25), individuals with the highest Mg intake was associated with a 95% decreased risk of glioma (OR = 0.06, 95% CI: 0.02–0.16), and individuals with the highest Fe intake was associated with a 93% decreased risk of glioma (OR = 0.07, 95% CI: 0.03–0.17), and individuals with the highest Zn intake was associated with an 89% decreased risk of glioma (OR = 0.07, 95% CI: 0.02–0.18), and individuals with the highest Cu intake was associated with an 87% decreased risk of glioma (OR = 0.09, 95% CI: 0.04–0.22).

**Table 3:** Adjusted ORs and 95% CIs for the association between dietary minerals and glioma.

	**T1**	**T2**	**T3**	**Continuous^c^**	***P_−*trend*_***
Ca	≤381.39	381.39–611.85	>611.85		
Case/control	211/127	187/157	108/222		
Model 1^a^	1	0.70 (0.51–0.97)	0.27 (0.19–0.39)	0.84 (0.80–0.88)	<0.001
Model 2^b^	1	0.46 (0.23–0.90)	0.11 (0.05–0.25)	0.65 (0.57–0.74)	<0.001
Mg	≤229.28	229.28–341.01	>341.01		
Case/control	191/147	197/140	118/219		
Model 1^a^	1	1.01 (0.74–1.38)	0.41 (0.30–0.57)	0.77 (0.70–0.84)	<0.001
Model 2^b^	1	0.45 (0.22–0.90)	0.06 (0.02–0.16)	0.18 (0.11–0.29)	<0.001
Fe	≤12.56	12.56–18.05	>18.05		
Case/control	184/154	200/137	122/215		
Model 1^a^	1	1.19 (0.87–1.63)	0.50 (0.37–0.68)	0.67 (0.57–0.80)	<0.001
Model 2^b^	1	0.35 (0.18–0.71)	0.07 (0.03–0.17)	0.04 (0.02–0.11)	<0.001
Zn	≤7.06	7.06–10.14	>10.14		
Case/control	191/147	176/161	139/198		
Model 1^a^	1	0.82 (0.61–1.11)	0.53 (0.39–0.73)	0.95 (0.93–0.98)	<0.001
Model 2^b^	1	0.32 (0.16–0.64)	0.07 (0.02–0.18)	0.62 (0.54–0.73)	<0.001
Cu	<1.08	1.08–1.67	>1.67		
Case/control	194/144	179/158	133/204		
Model 1^a^	1	0.83 (0.61–1.15)	0.49 (0.36–0.67)	0.76 (0.65–0.88)	<0.001
Model 2^b^	1	0.36 (0.18–0.71)	0.09 (0.04–0.22)	0.22 (0.13–0.39)	<0.001

T1, T2, and T3 represented the tertiles of mineral intake.

^a^ Model 1: Unadjusted model.

^b^Model 2: Adjusted for age, BMI, occupation, education level, household income, high-risk residential areas, smoking status, drinking status, history of allergies, history of head trauma, family history of cancer, physical activity, PUFAs intake, and energy intake.

^c^Ca, Mg per 100 mg/d increment, Fe per 10 mg/d increment, Zn, Cu per 1 mg/d increment.

The results of the analysis of the continuous variables showed that for each 100 mg/d increase in Ca intake, the risk of glioma decreased by 35% (OR = 0.65, 95% CI: 0.57–0.74), and for each 100 mg/d increase in Mg intake, the risk of glioma decreased by 82% (OR = 0.18, 95% CI: 0.11–0.29), and for each 10 mg/d increase in Fe intake, the risk of glioma decreased by 96% (OR = 0.04, 95% CI: 0.02–0.11), and for each 1 mg/d increase in Zn intake, the risk of glioma decreased by 38% (OR = 0.62, 95% CI: 0.54–0.73), and for each 1 mg/d increase in Zn intake, the risk of glioma decreased by 78% (OR = 0.22, 95% CI: 0.13–0.39).

### Minerals and pathological classification and grade of glioma

The analysis of pathological classifications of glioma showed that all five minerals were associated with decreased significantly risks of glioblastoma. The results were consistent with those of the overall population of gliomas. But for astrocytoma, the results of Fe and Cu were significant. Due to the small sample size of oligodendroglioma, no further analysis was carried out (Table 4).

**Table 4:** Adjusted ORs and 95% CIs for the association between dietary minerals and glioma of different pathological classifications.

**Pathological classification^c^**	**Model 1^a^**	***P*-value**	**Model 2^b^**	***P*-value**
**Astrocytoma**				
Ca	0.83 (0.75–0.92)	<0.001	0.01 (0.001–1.47)	0.072
Mg	0.76 (0.63–0.92)	0.004	0.002 (0.001–2.46)	0.086
Fe	0.62 (0.43–0.89)	0.011	–^*^	0.014
Zn	0.95 (0.89–1.01)	0.070	0.15 (0.01–1.67)	0.124
Cu	0.71 (0.52–0.98)	0.037	0.02 (0.001–0.43)	0.014
**Glioblastoma**				
Ca	0.85 (0.80–0.92)	<0.001	0.69 (0.53–0.89)	0.004
Mg	0.75 (0.65–0.87)	<0.001	0.14 (0.05–0.41)	<0.001
Fe	0.72 (0.57–0.92)	0.009	0.19 (0.05–0.64)	0.008
Zn	0.95 (0.91–0.99)	0.034	0.69 (0.53–0.88)	0.003
Cu	0.74 (0.59–0.92)	0.008	0.12 (0.03–0.47)	0.002

Due to the small sample size of oligodendroglioma, no further analysis was conducted.

^a^Model 1: Unadjusted model.

^b^Model 2: Adjusted for age, BMI, occupation, education level, household income, high-risk residential areas, smoking status, drinking status, history of allergies, history of head trauma, family history of cancer, physical activity, PUFAs intake, and energy intake.

^c^Ca, Mg per 100 mg/d increment, Fe per 10 mg/d increment, Zn, Cu per 1 mg/d increment.

^*^The lower limit of the confidence interval of the result was too small to be represented normally.

The results of minerals and different grades of gliomas showed that Mg, and Zn significantly were associated with a significantly decreased risk of low-grade gliomas. In contrast, the results of Ca, Fe, and Cu were not statistically significant. For high-grade gliomas, Ca, Mg, Fe, Zn, and Cu were associated with a significantly decreased risk (Table 5).

**Table 5:** Adjusted ORs and 95% CIs for the association between dietary minerals and glioma of different grades.

**Glioma grading^c^**	**Model 1^a^**	***P*-value**	**Model 2^b^**	***P*-value**
**Low grade**				
Ca	0.80 (0.70–0.90)	<0.001	0.02 (0.001–2.44)	0.111
Mg	0.77 (0.62–0.95)	0.014	0.01 (0.001–0.22)	0.006
Fe	0.58 (0.39–0.87)	0.008	–^*^	0.085
Zn	0.94 (0.88–1.01)	0.080	0.30 (0.12–0.77)	0.012
Cu	0.84 (0.61–1.14)	0.254	0.53 (0.13–2.22)	0.386
**High grade**				
Ca	0.86 (0.81–0.91)	<0.001	0.67 (0.55–0.81)	<0.001
Mg	0.78 (0.70–0.88)	<0.001	0.13 (0.06–0.30)	<0.001
Fe	0.74 (0.60–0.91)	0.003	0.09 (0.03–0.30)	<0.001
Zn	0.96 (0.93–0.99)	0.037	0.67 (0.55–0.82)	<0.001
Cu	0.79 (0.66–0.94)	0.009	0.12 (0.04–0.32)	<0.001

^a.^Model 1: Unadjusted model.

^b^Model 2: Adjusted for age, BMI, occupation, education level, household income, high-risk residential areas, smoking status, drinking status, history of allergies, history of head trauma, family history of cancer, physical activity, PUFAs intake, and energy intake.

^c^Ca, Mg per 100 mg/d increment, Fe per 10 mg/d increment, Zn, Cu per 1 mg/d increment.

^*^The lower limit of the confidence interval of the result was too small to be represented normally.

### Subgroup analysis

In the subgroup analysis by age, sex, BMI, occupation, education level, household income, smoking status, history of allergies, family history of cancer, and physical activity, we observed that most of the results in the subgroup analysis were consistent with the main results. Very few subgroups had no significant results due to the small sample size (Supplementary Table 8).

### Dose-response relationship

**Figure 1:**
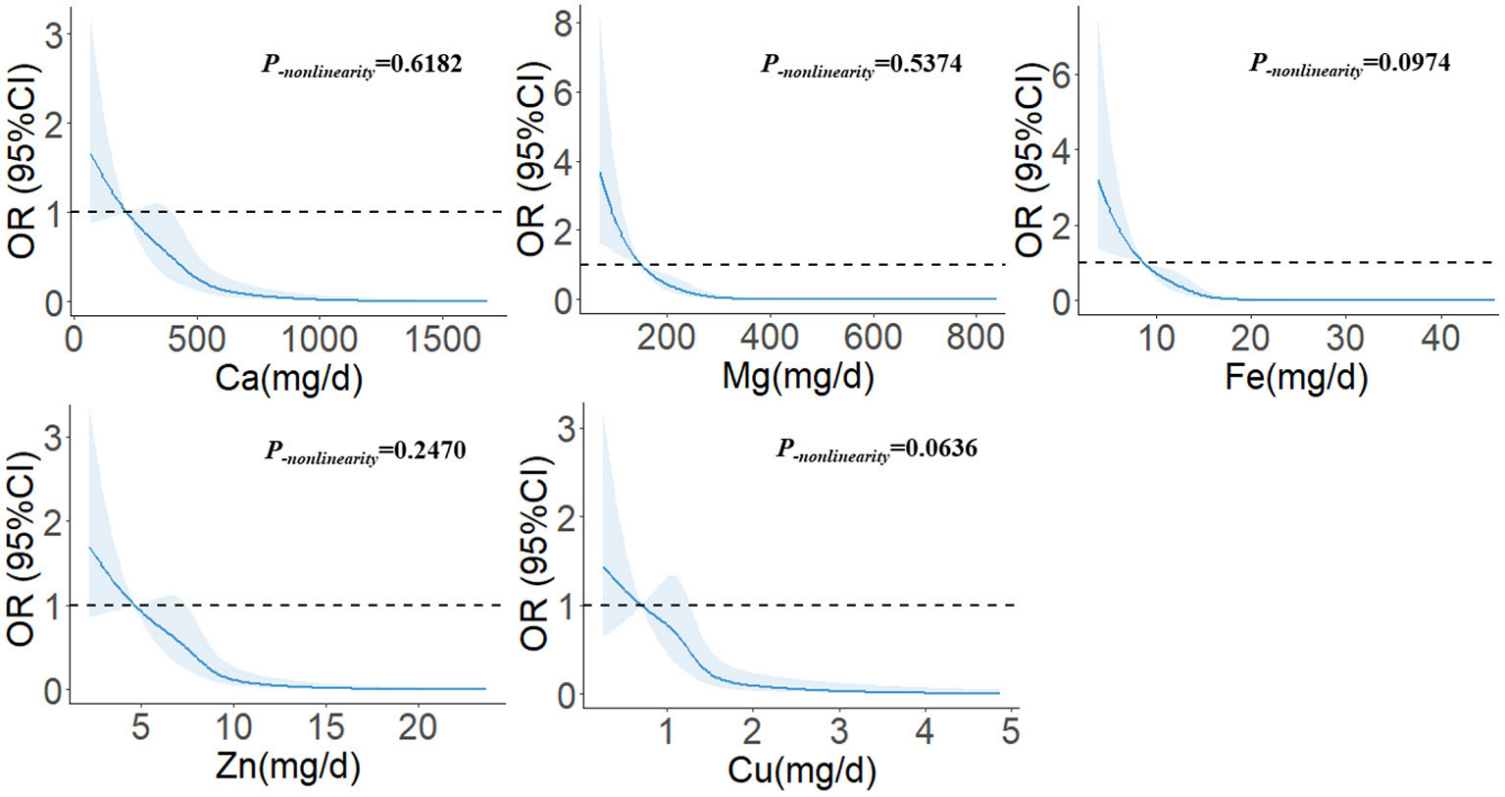
The restricted cubic spline for the associations between dietary minerals and glioma. The lines represent adjusted odds ratios based on restricted cubic splines for the intake in the regression model. Knots were placed at the 20th, 40th, 60th, and 80th percentiles of the dietary minerals intake, and the reference value was set at the 10th percentile. The adjusted factors were the same as in Model 2.

In Figure 1, we used restricted cubic splines to describe the relationship between minerals and the risk of glioma. There were linear dose-response relationships between the intakes of five minerals and the risk of glioma. For Ca, when the intake exceeded 398.02 mg/d, the risk of glioma decreased significantly with the increase in intake. When the intake exceeded 870.29 mg/d, the risk of glioma was relatively stable (*P*__−_nonlinearity_ = 0.6182). For Mg, when the intake exceeded 151.29 mg/d, the risk of glioma decreased significantly with the increase in intake. When the intake exceeded 310.40 mg/d, the risk of glioma was relatively stable (*P*_−nonlinearity_ = 0.5374). For Fe, when the intake exceeded 8.80 mg/d, the risk of glioma decreased significantly with the increase in intake. When the intake exceeded 17.55 mg/d, the risk of glioma was relatively stable (*P*_−nonlinearity_ = 0.0974). For Zn, when the intake exceeded 7.46 mg/d, the risk of glioma decreased significantly with the increase in intake. When the intake exceeded 12.55 mg/d, the risk of glioma was relatively stable (*P*_−nonlinearity_ = 0.2470). For Cu, when the intake exceeded 1.28 mg/d, the risk of glioma decreased significantly with the increase in intake. When the intake exceeded 2.65 mg/d, the risk of glioma was relatively stable (*P*_−nonlinearity_ = 0.0636).

## Discussion

Our study assessed the relationships between five common minerals and gliomas in the Chinese population. The results showed that the intakes of Ca, Mg, Fe, Zn, and Cu were significantly negatively associated with the risk of glioma. Similar results were observed in several subgroups, indicating that the association was relatively robust, especially in different pathological subtypes of gliomas and different grades of gliomas for the first time. It showed that this association was unlikely to be confused between different glioma subtypes. The restricted cubic spline model further confirmed a significant linear dose-response relationship between the five minerals and the risk of glioma, and with the increase in intake, the risk of glioma tended to be stable.

Ca is the most abundant mineral element in the human body, of which about 99% is concentrated in bones and teeth and plays a crucial role in bone mineralization and a wide range of biological functions (31). As an essential element for the human body, people can only get it from Ca-rich food sources, including milk and soybeans (31). Based on the physiological effects of Ca, studies showed that it was closely related to osteoporosis and cardiovascular disease, and cancer was no exception (32). In contrast, studies on dietary Ca and glioma were rare. Yekta et al. found a significant negative association between dietary Ca intakes in 128 glioma patients and 256 healthy individuals in a case-control study based on an Iranian hospital (OR = 0.23, 95% CI: 0.08–0.65) (17). Due to differences in dietary Ca sources between the Chinese population and the Middle East, although Ca intake in this study was higher than ours, our study found similar results. Higher dietary Ca intake was associated with a significantly decreased risk of glioma (OR = 0.11, 95% CI: 0.05–0.25) with a significant linear-dose-response relationship, which complemented the evidence for low Ca intake. For glioma subtypes, dietary Ca also had the same protective effect against glioblastoma (OR = 0.69, 95% CI: 0.53–0.89). This was similar to earlier results from the San Francisco Bay Area Adult Glioma Study. Tedeschi-Blok et al. compared dietary Ca intakes in 337 astrocytoma patients and 450 controls and found that dietary Ca intake was inversely associated with astrocytomas in a female-only population. Ca in our study population was inversely associated with astrocytoma, and the Ca intake of this population was closer to our study (18). In addition, a meta-analysis of the dose-response relationship showed that each 100 mg/d increase in Ca intake reduced the risk of glioma by 7% (OR = 0.93, 95% CI: 0.88–0.98), which was similar to our results. However, this meta-analysis included only four studies with high heterogeneity, and the results still needed to be further confirmed in the future (33). Most of its mechanisms were currently considered to be related to the regulation of parathyroid hormone. Increased Ca levels in the body can reduce the release of parathyroid hormone (34), which was thought to play a promoting role in the development of cancer (35, 36). In gliomas, parathyroid hormone-related proteins were also found to regulate the transcriptional activation of glioma-related oncogenes (37), and immunohistochemical results showed that parathyroid hormone-related proteins were present in astrocytomas, suggesting that parathyroid hormone-related proteins may be related to the imbalance of growth or differentiation of astrocytoma cells (38). In addition, intracellular Ca and Ca signal pathways were closely related to gliomas (19). Elevated intracellular Ca^2+^ can activate nitric oxide synthase to generate nitric oxide, which impacted tumorigenesis (39), but it was difficult to directly link dietary Ca intake with Ca signaling channels.

Fe is involved in various metabolic processes in the body, including oxygen transport, DNA synthesis, and electron transport, and is an essential element in almost all organisms (40). There are two main dietary Fe forms—heme Fe and non-heme Fe (41). Among them, heme Fe mainly comes from meat. In the western diet, heme Fe accounted for 10% of the total dietary Fe, but because the body more easily absorbed it, it accounted for nearly 2/3 of Fe absorption (42). Non-heme Fe exists mainly in plants. Nutritional disorders caused by Fe deficiency, such as anemia, infection, liver disease, and nervous system disease, have become public health issues of great concern (43). There have been many reports of dietary Fe and cancer in recent years. But studies on Fe and glioma were rare. Parent et al. found a non-significant association between Fe in occupational exposure and glioma in a population-based multicenter case-control study (OR = 1.10, 95% CI: 0.80–1.50). Still, the exposure route in this study was mainly the respiratory system (44). Ward et al. followed up for 14.1 years in the European Prospective Investigation into Cancer and Nutrition and found that total dietary Fe (HR = 0.94, 95% CI: 0.71–1.24) and heme Fe (HR = 0.96, 95% CI: 0.73–1.26) were not associated with the risk of glioma (20). This was not consistent with our results. We found that higher dietary Fe intake had a protective effect against gliomas (OR = 0.07, 95% CI: 0.03–0.17), and similar results were observed in astrocytomas and glioblastomas as well as in high-grade gliomas. In addition, because Fe was not only an essential element of the body but also potentially toxic to cells, it was significant to describe its dose-response relationship. We found a linear dose-response relationship between dietary Fe and glioma, and when the intake exceeded 17.55 mg/d, the risk of glioma did not change (*P*_−nonlinearity_ = 0.0974). Although dietary Fe has not shown a protective effect against gliomas in previous studies, studies on internal exposure seemed to support our results. Anic et al. determined the toenail Fe concentration by neutron activation analysis and found a significant negative association between toenail Fe and the risk of glioma (OR = 0.42, 95% CI: 0.19–0.95) (21). The relationship and mechanism between Fe and glioma were relatively complex. Bioinformatics studies have found that Fe metabolism-related genes can be used as prognostic indicators of low-grade gliomas (45). Some studies also found that Fe played an essential role in treating gliomas. In animal experiments, it was found that after intravenous injection of Fe complex into male nude mice with glioma, the tumor growth of nude mice was significantly inhibited after 3 weeks, and the possible mechanism was apoptosis (46). Eales et al. also found that verteporfin can radically and selectively kill anoxic glioma cells by binding free Fe (47).

Zn is the second most abundant transition metal ion in organisms, second only to Fe, and is indispensable to the growth and development of plants, animals, and microorganisms (48, 49). It can not only be used as a cofactor of more than 300 enzymes (50) but also play a key role in oxidative stress, immunity, and aging (49), and it has been reported that dietary Zn has protective effects against depression, type 2 diabetes and some cancers (51). Dietary Zn reduced the risk of cancer, mainly in the digestive system (52, 53). There have been few studies on Zn and gliomas. Dimitropoulou et al. observed only a slight protective effect of dietary Zn against meningioma (OR = 0.62, 95% CI: 0.39–0.99) in the study of adult brain tumors in the UK, but the association with glioma was not significant (OR = 0.92, 95% CI: 0.66–1.28) (23). However, in our study, higher dietary Zn intake significantly reduced the risk of glioma (OR = 0.07, 95% CI: 0.02–0.18), and there was a significant linear-dose response relationship. When the intake was 7.46–12.55 mg/d, the risk of glioma decreased with increased intake. The risk did not change beyond 12.55 mg/d. The mechanism of Zn involved in the development of glioma may be various. On the one hand, Zn, as a component of superoxide dismutase (54), had a strong antioxidant effect and played an essential role in oxidative stress and repairing DNA damage (49, 55). Along with the depletion of Zn in the body, this can lead to DNA damage and the production of free radicals, which can lead to the formation of tumors (56, 57). This was no exception in glioma (58). On the other hand, appropriate Zn can induce apoptosis of glioma cells. Haase et al. found in C6 rat glioma cells that Zn can promote proliferation and growth at a concentration of 50–100 μM, but too low (<50 μM) or too high (>200 μM) can induce apoptosis, especially when it exceeded 300 μM, it seemed to cause the necrosis of glioma cells (59). In addition, Zn may act as an epigenetic regulator of gliomas by promoting proper DNA folding, protecting genetic material from oxidative damage, and controlling the activation of enzymes involved in epigenetic regulation (60).

Mg is the fourth most abundant mineral in the body and the second most abundant intracellular divalent cation (61, 62). Whole grains, green vegetables, and nuts are rich dietary sources of Mg, but the loss of Mg during cooking and processing partly explains Mg deficiency (63). Because Mg is involved in many biological processes in the body, including energy production, glycolysis, oxidative phosphorylation, nucleic acid, and protein synthesis (62, 64), it was closely related to muscle health, asthma, cardiovascular disease, and mental illness (62). But the impact on cancer was currently inconsistent. In animal models, low Mg status was found to have a dual effect against tumors—inhibiting primary tumor growth and promoting metastatic tumor engraftment (65). However, epidemiological evidence still suggested that Mg deficiency may increase the risk of certain cancers. The results of an earlier meta-analysis showed that higher dietary Mg intake was associated with a significant reduction in overall cancer risk (RR = 0.80, 95% CI: 0.66–0.97) (66). Our study provided some evidence of the association between dietary Mg and gliomas, with higher dietary Mg having a protective effect against gliomas (OR = 0.06, 95% CI: 0.02–0.16), especially in low-grade gliomas. Mg has shown some anti-inflammatory effects in preclinical and epidemiological studies. Both dietary Mg intake and serum Mg levels were associated with increased levels of low-grade systemic inflammation, pro-inflammatory factors, and inflammatory markers (62, 67–69). This may create a microenvironment that was conducive to tumor invasion and metastasis (70). Since the presence of inflammatory cells and the release of inflammatory mediators also promoted glioma proliferation, angiogenesis, and attack, it seemed impossible to ignore the effect of Mg on gliomas (71). In addition, it may also be related to Mg transporter 1, which was highly selective for Mg transport (72). Recent studies have also found that the overexpression of Mg transporter 1 promoted the growth of glioma cells through the up-regulation of PD-L1 expression mediated by the ERK/MAPK signaling pathway (73), but whether dietary Mg was involved remains to be further explored.

Although Cu is also an essential trace element for the body, compared with the previous minerals, the demand for Cu is deficient, with only about 100 mg of Cu in the human body (74). Animal offal, corn products, certain vegetables, and individual fruits are good sources of dietary Cu (74). Since Cu was a cofactor for many oxidoreductases, it was involved in the body's antioxidant defense, neuropeptide synthesis, and immune function (75, 76). It also played an important role in fetal development (77), cardiovascular disease, and cognitive function (74). Studies on Cu and cancer were rare, and most studies have found no significant association between dietary Cu and some cancers. However, our study found that higher dietary Cu significantly reduced the risk of glioma (OR = 0.09, 95% CI: 0.04–0.22). Still, its effect was not as significant as that of the other four minerals. Moreover, dietary Cu seemed to have a protective effect only on high-grade gliomas (OR = 0.12, 95% CI: 0.04–0.32). No similar result was observed in low-grade gliomas (OR = 0.53, 95% CI: 0.13–2.22). Since both Cu deficiency and Cu excess were harmful to health, some scholars proposed that the dose-response curve between Cu and health was *U*-shaped (78). This was similar to the results of our study. Although there was a linear dose-response relationship between dietary Cu and glioma in this study, the risk leveled off when the intake exceeded a certain level. Due to the relatively narrow range of dietary Cu intake, we suspected that the current dose-response curve might be the left half of the *U*-shaped curve. In addition, some studies also found that Cu played a role in the treatment of glioma. *In vitro* studies, Trejo-Solis et al. found that Cu compounds induced autophagy and apoptosis of glioma cells by increasing the production of intracellular reactive oxygen species and the activity of c-junNH2-terminal kinase (79). Castillo-Rodriguez et al. found anti-proliferation, pro-apoptosis, and anti-invasion effects of Cu coordination compounds on U373 human glioma cells and significantly reduced tumor volume, cell proliferation, and mitotic index in mice transplanted with U373 glioma cells, and apoptosis index was increased (80).

The limitation of this study was that we could not explore the association between different forms and valence minerals in diet and glioma. Since the food composition table only provided the total amount of minerals without differentiating their form and valence, different forms and valence of minerals may have different effects on the body, we cannot analyze the results of the form and valence of these minerals on glioma in detail. Secondly, we only evaluated dietary sources for the relationship between minerals and glioma, and we could not comprehensively assess other sources, such as air. However, the sources of minerals were mainly dietary, so the results of this study were still of certain significance. In addition, because the study was a case-control study, we could not verify the causal relationship between the two and avoid inherent bias. In order to reduce the impact of information bias on this study, all questionnaires were completed face to face by investigators with medical education background. These investigators can participate in the survey only after receiving unified training and strict assessment before conducting the survey. In addition, in order to improve the accuracy of the dietary survey, the investigators assisted participants in estimating the amount of food in detail through food picture flip books containing different food volumes and qualities. However, the study still had some advantages. First, we explored the association between five common dietary minerals and gliomas. The results were consistent with existing *in-vitro* studies, especially for Cu and Mg, which lacked clinical studies and explored the association between gliomas of different pathological subtypes and pathological grades and minerals. Moreover, this was the first time that these dose-response relationships between dietary mineral intake and the risk of glioma were described, and the significant linear dose-response relationships provided further population evidence for mineral prevention and treatment of glioma.

## Conclusion

In summary, we observe that higher intakes of Ca, Mg, Fe, Zn, and Cu were associated with a decreased risk of glioma. Therefore, we may not be able to ignore the influence of dietary minerals on glioma. In the future, further prospective studies should be conducted to verify their relationship.

## Data availability statement

The original contributions presented in the study are included in the article/Supplementary material, further inquiries can be directed to the corresponding author.

## Ethics statement

The studies involving human participants were reviewed and approved by the Institutional Review Board of Beijing Tiantan Hospital, Capital Medical University (No. KY2022-203-02). The patients/participants provided their written informed consent to participate in this study.

## Author contributions

WL and WZ contributed to the conception or design of the work and wrote the manuscript. WZ, YH, XK, CW, and FC contributed to data collection and analysis. ZK, SY, RZ, and YP contributed to data collection and management. All authors have read and approved the final manuscript.
