# Supplementary material for: Association between dietary minerals and glioma: A case-control study based on Chinese population
Source: Front Nutr. 2023 Mar 2;10:1118997. doi: 10.3389/fnut.2023.1118997 (PMC10018027; doi:10.3389/fnut.2023.1118997)
Supplement: Supplementary file 1 [file Data_Sheet_1.docx]

Supplementary Material

Table S1. Food items in the food frequency questionnaire.

Table S2. Reproducibility of food consumption in food frequency questionnaire.

Table S3. Reproducibility of nutrients intake in food frequency questionnaire.

Table S4. Validity of food consumption in food frequency questionnaire.

Table S5. Validity of nutrients intake in food frequency questionnaire.

Table S6. Dietary intakes of study participants.

Table S7. Correlations between dietary minerals among the case-control participants.

Table S8. Adjusted ORs and 95% CIs for gliomas in subgroups.

Figure S1. The flow chart of the study population.

Table S1. Food items in the food frequency questionnaire.

| Food group (n) | Items |
| --- | --- |
| Refined grains (9) | Rice, steamed buns, scones, porridge, deep-fried dough sticks, noodles, instant noodles, vermicelli, dumplings |
| Whole grains (2) | Corn，millet and oats |
| Tubers (2) | Potatoes, sweet potatoes |
| Legume and Products (5) | Soy, soy milk, tofu, other soy products, other legumes |
| Vegetables (23) | Green leafy vegetables, kale, cabbage, celery, eggplant, cucumber, white gourd, pumpkin, carrot, radish, green shoot, broccoli, bell pepper, pepper, tomato, lotus root, chive, garlic moss, bitter melon, onion, green bean, zucchini, cauliflower |
| Fungi and algae (4) | Mushrooms, Auricularia auricula, tremella, kelp |
| Fruits (20) | Apple, pear, orange, banana, peach, watermelon, melon, grape, pineapple, kiwi fruit, strawberries, cherries, blueberries, mulberries, shaddock, hawthorn, pitaya, Chinese date, mango, cherry tomato |
| Red meat (4) | Pork (muscle), pork (fat & muscle), beef, mutton |
| Poultry (2) | Chicken, duck |
| Animal viscera (4) | Liver, blood, intestine, other viscera |
| Fish and seafood (5) | Freshwater fish, sea fish, shrimp, crab, mollusk |
| Egg (1) | Fresh egg |
| Dairy products (4) | Milk, yogurt, milk powder, cheese |
| Nuts (4) | Peanuts, sunflower seeds, walnuts, other nuts |
| Sweet food (5) | Bread, honey, biscuit, cake, Chinese pastry |
| Sugary drink (3) | Carbonated drinks, fruit and vegetable drinks, functional drinks |
| Tea and coffee (3) | Green tea, black tea, coffee |
| Condiment (4) | Salt, animal oil, olive oil, other vegetable oil |
| Curing food (3) | Sauerkraut, pickle, fermented bean curd |
| Processed products (3) | Bacon, sausage and other smoked meat, salted duck eggs, preserved eggs |
| Alcohol (4) | Beer, spirit, wine, rice wine |

Table S2. Reproducibility of food consumption in food frequency questionnaire.

| Food group | FFQ1 | FFQ2 | *P-value* | Correlation Coefficient |
| --- | --- | --- | --- | --- |
| Grains(g/d) | 153.06±62.60 | 167.79±51.88 | 0.325 | 0.502^**^ |
| Tubers(g/d) | 53.86±105.32 | 48.93±86.46 | 0.807 | 0.604^**^ |
| Legume and Products(g/d) | 10.60±12.92 | 13.92±14.40 | 0.264 | 0.720^**^ |
| Vegetables(g/d) | 351.39±251.83 | 305.16±184.29 | 0.420 | 0.845^**^ |
| Fruits(g/d) | 265.16±180.62 | 257.05±193.98 | 0.739 | 0.641^**^ |
| Red meat(g/d) | 100.07±98.99 | 95.67±88.47 | 0.994 | 0.549^**^ |
| Poultry(g/d) | 13.31±12.40 | 11.30±10.35 | 0.624 | 0.847^**^ |
| Fish and seafood(g/d) | 18.25±22.38 | 25.99±37.54 | 0.501 | 0.590^**^ |
| Egg(g/d) | 50.51±29.05 | 50.67±26.06 | 0.807 | 0.633^**^ |
| Dairy products(g/d) | 160.43±131.44 | 144.85±112.39 | 0.750 | 0.623** |
| Sweet food(g/d) | 29.25±35.42 | 24.97±31.13 | 0.716 | 0.683^**^ |
| Curing and processed products(g/d) | 19.44±24.36 | 17.27±20.20 | 0.923 | 0.741^**^ |

Note:T-test and Person correlation coefficient were used for cereals and vegetables, and Mann-Whitney test and Spearman correlation coefficient were used for other food groups.

**P*<0.05，***P*<0.01

Table S3. Reproducibility of nutrients intake in food frequency questionnaire.

| Nutrients | FFQ1 | FFQ2 | *P-value* | Correlation Coefficient |
| --- | --- | --- | --- | --- |
| Energy(kcal/d) ^a^ | 1769.24±580.51 | 1787.20±509.83 | 0.451 | 0.555^**^ |
| Carbohydrate (g/d) ^b^ | 207.74±85.40 | 217.64±79.41 | 0.644 | 0.640^**^ |
| Protein(g/d) ^a^ | 70.95±23.80 | 71.67±26.77 | 0.679 | 0.608^**^ |
| Fat(g/d) ^a^ | 76.08±26.09 | 73.21±17.35 | 0.802 | 0.437^*^ |
| Cholesterol(mg/d) ^b^ | 508.36±204.70 | 501.79±211.69 | 0.903 | 0.645^**^ |
| Dietary fiber(g/d) ^b^ | 9.42±4.51 | 10.06±5.37 | 0.619 | 0.706^**^ |
| Carotene(μg/d) ^a^ | 2421.98±1542.63 | 2562.50±1524.28 | 0.701 | 0.646^**^ |
| Vitamin A(μg/d) ^a^ | 641.85±573.83 | 768.04±794.33 | 0.569 | 0.488^**^ |
| Vitamin B1(mg/d) ^a^ | 0.87±0.37 | 0.89±0.42 | 0.813 | 0.675^**^ |
| Vitamin B2(mg/d) ^b^ | 0.92±0.31 | 0.92±0.33 | 0.995 | 0.696^**^ |
| Vitamin C(mg/d) ^a^ | 125.23±75.97 | 122.85±57.80 | 0.906 | 0.629^**^ |
| Vitamin E(mg/d) ^a^ | 12.70±6.52 | 13.77±7.35 | 0.535 | 0.694^**^ |
| Nicotinic acid(mg/d) ^a^ | 16.65±7.13 | 15.48±6.38 | 0.636 | 0.619^**^ |
| Folate(μg/d) ^b^ | 280.96±100.12 | 291.34±115.10 | 0.711 | 0.768^**^ |
| Sodium(mg/d) ^b^ | 4919.91±361.80 | 4952.62±406.97 | 0.743 | 0.460^*^ |
| Calcium(mg/d) ^b^ | 540.27±250.19 | 541.86±224.01 | 0.979 | 0.807^**^ |
| Magnesium(mg/d) ^b^ | 286.28±110.27 | 299.07±110.45 | 0.655 | 0.756^**^ |
| Iron(mg/d) ^a^ | 16.82±6.33 | 17.70±6.93 | 0.442 | 0.668^**^ |
| Zinc(mg/d) ^a^ | 9.97±3.63 | 10.37±4.16 | 0.595 | 0.487^**^ |
| Copper(mg/d) ^b^ | 1.44±0.54 | 1.63±0.79 | 0.273 | 0.613^**^ |

a. Mann-Whitney test and Spearman correlation coefficient were used for these nutrients.

b. T-test and Person correlation coefficient were used for these nutrients.

**P*<0.05，***P*<0.01

Table S4. Validity of food consumption in food frequency questionnaire.

| Food group | FFQ1 | 24-hour Recall | *P-value* | Correlation Coefficient |
| --- | --- | --- | --- | --- |
| Grains(g/d) | 153.06±62.60 | 163.07±56.65 | 0.519 | 0.743^**^ |
| Tubers(g/d) | 53.86±105.32 | 43.97±41.47 | 0.699 | 0.562^**^ |
| Legume and Products(g/d) | 10.60±12.92 | 6.49±5.83 | 0.167 | 0.549^**^ |
| Vegetables(g/d) | 351.39±251.83 | 236.64±139.89 | 0.033 | 0.702^**^ |
| Fruits(g/d) | 265.16±180.62 | 142.00±143.99 | 0.003 | 0.471^**^ |
| Red meat(g/d) | 100.07±98.99 | 76.27±56.45 | 0.745 | 0.594^**^ |
| Poultry(g/d) | 13.31±12.40 | 22.91±27.19 | 0.306 | 0.779^**^ |
| Fish and seafood(g/d) | 18.25±22.38 | 13.56±24.68 | 0.036 | 0.490^**^ |
| Egg(g/d) | 50.51±29.05 | 57.13±34.15 | 0.332 | 0.381^*^ |
| Dairy products(g/d) | 160.43±131.44 | 83.43±114.30 | 0.007 | 0.412^*^ |
| Sweet food(g/d) | 29.25±35.42 | 26.00±51.37 | 0.056 | 0.396^*^ |
| Curing and processed products(g/d) | 19.44±24.36 | 7.57±12.59 | 0.011 | 0.560^**^ |

Note: T-test and Person correlation coefficient were used for cereals and vegetables, and Mann-Whitney test and Spearman correlation coefficient were used for other food groups.

**P*<0.05，***P*<0.01

Table S5. Validity of nutrients intake in food frequency questionnaire.

| Nutrients | FFQ1 | 24-hour Recall | *P-value* | Correlation Coefficient |
| --- | --- | --- | --- | --- |
| Energy(kcal/d) ^a^ | 1769.24±580.51 | 1638.24±338.19 | 0.988 | 0.503^**^ |
| Carbohydrate(g/d) ^b^ | 207.74±85.40 | 185.38±40.60 | 0.202 | 0.380^*^ |
| Protein(g/d) ^a^ | 70.95±23.80 | 61.98±16.99 | 0.160 | 0.430^*^ |
| Fat(g/d) ^a^ | 76.08±26.09 | 74.69±19.53 | 0.813 | 0.707^**^ |
| Cholesterol(mg/d) ^a^ | 508.36±204.70 | 517.17±230.45 | 0.929 | 0.476^**^ |
| Dietary fiber(g/d) ^b^ | 9.42±4.51 | 5.48±1.90 | <0.001 | 0.724^**^ |
| Carotene(μg/d) ^a^ | 2421.98±1542.63 | 2682.04±2182.24 | 0.965 | 0.682^**^ |
| Vitamin A(μg/d) ^a^ | 641.85±573.83 | 486.50±226.53 | 0.261 | 0.489^**^ |
| Vitamin B1(mg/d) ^a^ | 0.87±0.37 | 0.71±0.27 | 0.086 | 0.603^**^ |
| Vitamin B2(mg/d) ^b^ | 0.92±0.31 | 0.68±0.22 | 0.001 | 0.447^*^ |
| Vitamin C(mg/d )^a^ | 125.23±75.97 | 85.06±41.92 | 0.021 | 0.502^**^ |
| Vitamin E(mg/d) ^a^ | 12.70±6.52 | 7.24±3.15 | <0.001 | 0.679^**^ |
| Nicotinic acid(mg/d) ^a^ | 16.65±7.13 | 13.80±4.61 | 0.139 | 0.444^*^ |
| Folate(μg/d) ^b^ | 280.96±100.12 | 264.73±119.39 | 0.571 | 0.645^**^ |
| Sodium(mg/d) ^b^ | 4919.91±361.80 | 4666.45±268.48 | 0.003 | 0.509^**^ |
| Calcium(mg/d) ^b^ | 540.27±250.19 | 462.81±222.01 | 0.210 | 0.804^**^ |
| Magnesium(mg/d) ^b^ | 286.28±110.27 | 212.34±70.45 | 0.003 | 0.563^**^ |
| Iron(mg/d) ^a^ | 16.82±6.33 | 15.87±5.04 | 0.756 | 0.731^**^ |
| Zinc(mg/d) ^a^ | 9.97±3.63 | 9.15±3.01 | 0.515 | 0.680^**^ |
| Copper(mg/d) ^b^ | 1.44±0.54 | 0.95±0.32 | <0.001 | 0.512^**^ |

a. Mann-Whitney test and Spearman correlation coefficient were used for these nutrients.

b. T-test and Person correlation coefficient were used for these nutrients.

**P*<0.05，***P*<0.01

Table S6. Dietary intakes of study participants.

| Food group | Case | Control | *P-value* |
| --- | --- | --- | --- |
| Refined grains(g/d) | 202.31±93.36 | 155.37±89.63 | <0.001 |
| Whole grains(g/d) | 7.61±14.13 | 15.13±23.76 | <0.001 |
| Legume and products(g/d) | 8.78±10.43 | 15.02±19.63 | <0.001 |
| Tubers(g/d) | 28.20±43.42 | 46.43±58.95 | <0.001 |
| Vegetables(g/d) | 219.08±155.89 | 409.05±245.67 | <0.001 |
| Fungi and algae(g/d) | 15.72±20.11 | 29.66±31.59 | <0.001 |
| Fruits(g/d) | 167.10±152.56 | 249.70±191.21 | <0.001 |
| Red meat(g/d) | 64.39±54.82 | 63.46±51.64 | 0.780 |
| Poultry(g/d) | 14.76±18.94 | 14.45±19.89 | 0.803 |
| Fish and seafood(g/d) | 14.04±22.56 | 24.45±33.66 | <0.001 |
| Egg(g/d) | 51.10±34.96 | 48.54±34.79 | 0.243 |
| Dairy products(g/d) | 131.43±156.82 | 153.98±162.14 | 0.025 |
| Nuts(g/d) | 10.85±21.17 | 9.95±15.33 | 0.441 |
| Sweet food(g/d) | 21.69±41.16 | 22.81±32.87 | 0.632 |
| Curing and Processed products(g/d) | 12.77±18.02 | 13.56±23.55 | 0.553 |
| Alcohol(g/d) | 14.61±37.12 | 5.46±17.76 | <0.001 |

Table S7. Correlations between dietary minerals among the case-control participants.

|  | Ca | Mg | Fe | Zn | Cu |
| --- | --- | --- | --- | --- | --- |
| Ca | 1.000 | 0.807 | 0.719 | 0.709 | 0.728 |
| Mg |  | 1.000 | 0.924 | 0.844 | 0.914 |
| Fe |  |  | 1.000 | 0.919 | 0.888 |
| Zn |  |  |  | 1.000 | 0.820 |
| Cu |  |  |  |  | 1.000 |

Note: *P* values of all correlation coefficients were less than 0.01.

Table S8. Adjusted ORs and 95% CIs for gliomas in subgroups.

| Subgroup^a^ | Model 1^b^ | *P-value* | Model 2^c^ | *P-value* |
| --- | --- | --- | --- | --- |
| Age |  |  |  |  |
| ≤40(n=500) |  |  |  |  |
| Ca | 0.88(0.83-0.94) | <0.001 | 0.72(0.64-0.81) | <0.001 |
| Mg | 0.87(0.77-0.98) | 0.026 | 0.28(0.19-0.42) | <0.001 |
| Fe | 0.78(0.62-1.00) | 0.051 | 0.03(0.01-0.08) | <0.001 |
| Zn | 0.98(0.94-1.02) | 0.387 | 0.69(0.61-0.79) | <0.001 |
| Cu | 0.91(0.75-1.12) | 0.387 | 0.26(0.15-0.44) | <0.001 |
| ＞41(n=512) |  |  |  |  |
| Ca | 0.81(0.75-0.86) | <0.001 | 0.64(0.56-0.73) | <0.001 |
| Mg | 0.67(0.59-0.77) | <0.001 | 0.21(0.14-0.31) | <0.001 |
| Fe | 0.58(0.46-0.74) | <0.001 | 0.07(0.03-0.16) | <0.001 |
| Zn | 0.93(0.89-0.97) | 0.001 | 0.71(0.63-0.80) | <0.001 |
| Cu | 0.64(0.52-0.78) | <0.001 | 0.32(0.20-0.51) | <0.001 |
| Sex |  |  |  |  |
| Male(n=568) |  |  |  |  |
| Ca | 0.86(0.81-0.91) | <0.001 | 0.62(0.56-0.70) | <0.001 |
| Mg | 0.81(0.72-0.90) | <0.001 | 0.19(0.13-0.28) | <0.001 |
| Fe | 0.79(0.64-0.97) | 0.021 | 0.06(0.03-0.13) | <0.001 |
| Zn | 0.98(0.95-1.02) | 0.296 | 0.74(0.67-0.82) | <0.001 |
| Cu | 0.81(0.67-0.98) | 0.026 | 0.20(0.12-0.33) | <0.001 |
| Female(n=444) |  |  |  |  |
| Ca | 0.82(0.76-0.88) | <0.001 | 0.78(0.68-0.90) | 0.001 |
| Mg | 0.71(0.61-0.83) | <0.001 | 0.31(0.20-0.49) | <0.001 |
| Fe | 0.51(0.37-0.69) | <0.001 | 0.04(0.01-0.12) | <0.001 |
| Zn | 0.90(0.86-0.95) | <0.001 | 0.66(0.56-0.78) | <0.001 |
| Cu | 0.71(0.57-0.89) | 0.003 | 0.41(0.24-0.72) | 0.002 |
| BMI |  |  |  |  |
| ≤23.31(n=506) |  |  |  |  |
| Ca | 0.82(0.76-0.87) | <0.001 | 0.72(0.64-0.82) | <0.001 |
| Mg | 0.76(0.66-0.87) | <0.001 | 0.32(0.22-0.47) | <0.001 |
| Fe | 0.67(0.52-0.86) | 0.002 | 0.15(0.07-0.34) | <0.001 |
| Zn | 0.93(0.89-0.98) | 0.003 | 0.71(0.62-0.82) | <0.001 |
| Cu | 0.74(0.59-0.91) | 0.005 | 0.45(0.28-0.72) | 0.001 |
| ＞23.31(n=506) |  |  |  |  |
| Ca | 0.86(0.81-0.92) | <0.001 | 0.67(0.59-0.76) | <0.001 |
| Mg | 0.76(0.67-0.86) | <0.001 | 0.21(0.14-0.31) | <0.001 |
| Fe | 0.64(0.50-0.81) | <0.001 | 0.02(0.01-0.06) | <0.001 |
| Zn | 0.96(0.92-1.00) | 0.048 | 0.70(0.62-0.78) | <0.001 |
| Cu | 0.76(0.62-0.93) | 0.007 | 0.20(0.12-0.34) | <0.001 |
| Occupation |  |  |  |  |
| Manual workers(n=237) |  |  |  |  |
| Ca | 0.83(0.76-0.92) | <0.001 | 0.67(0.54-0.81) | <0.001 |
| Mg | 0.77(0.64-0.92) | 0.003 | 0.28(0.15-0.51) | <0.001 |
| Fe | 0.62(0.44-0.87) | 0.005 | 0.04(0.01-0.17) | <0.001 |
| Zn | 0.95(0.89-1.01) | 0.071 | 0.64(0.52-0.78) | <0.001 |
| Cu | 0.70(0.52-0.93) | 0.014 | 0.44(0.22-0.87) | 0.018 |
| Mental workers(n=571) |  |  |  |  |
| Ca | 0.88(0.83-0.93) | <0.001 | 0.70 (0.62-0.78) | <0.001 |
| Mg | 0.84(0.75-0.94) | 0.002 | 0.21(0.14-0.30) | <0.001 |
| Fe | 0.79(0.63-0.98) | 0.029 | 0.04(0.02-0.09) | <0.001 |
| Zn | 0.98(0.94-1.02) | 0.307 | 0.74(0.67-0.83) | <0.001 |
| Cu | 0.88(0.73-1.05) | 0.162 | 0.21(0.12-0.35) | <0.001 |
| Others(n=204) |  |  |  |  |
| Ca | 0.76(0.67-0.86) | <0.001 | 0.64(0.49-0.84) | 0.001 |
| Mg | 0.56(0.44-0.71) | <0.001 | 0.22(0.11-0.43) | <0.001 |
| Fe | 0.44(0.28-0.69) | <0.001 | 0.10(0.02-0.40) | 0.001 |
| Zn | 0.87(0.80-0.95) | 0.001 | 0.54(0.40-0.73) | <0.001 |
| Cu | 0.54(0.37-0.78) | 0.001 | 0.22(0.08-0.56) | 0.002 |
| Education level |  |  |  |  |
| Middle school and below(n=385)^d^ |  |  |  |  |
| Ca | 0.82(0.77-0.89) | <0.001 | 0.66(0.56-0.78) | <0.001 |
| Mg | 0.72(0.62-0.83) | <0.001 | 0.30(0.19-0.47) | <0.001 |
| Fe | 0.57(0.43-0.76) | <0.001 | 0.04(0.02-0.12) | <0.001 |
| Zn | 0.93(0.88-0.98) | 0.003 | 0.64(0.55-0.76) | <0.001 |
| Cu | 0.68(0.54-0.86) | 0.001 | 0.40(0.23-0.69) | 0.001 |
| University and above(n=627) |  |  |  |  |
| Ca | 0.87(0.82-0.92) | <0.001 | 0.72(0.65-0.80) | <0.001 |
| Mg | 0.79(0.70-0.89) | <0.001 | 0.24(0.17-0.34) | <0.001 |
| Fe | 0.74(0.59-0.91) | 0.005 | 0.07(0.03-0.14) | <0.001 |
| Zn | 0.97(0.94-1.01) | 0.162 | 0.75(0.68-0.83) | <0.001 |
| Cu | 0.81(0.67-0.98) | 0.033 | 0.24(0.15-0.39) | <0.001 |
| Household income |  |  |  |  |
| <3,000 ¥/month(n=141) |  |  |  |  |
| Ca | 0.94(0.84-1.05) | 0.242 | 0.68(0.51-0.91) | 0.008 |
| Mg | 1.02(0.82-1.27) | 0.852 | 0.38(0.17-0.85) | 0.018 |
| Fe | 0.97(0.62-1.50) | 0.885 | 0.04(0.01-0.33) | 0.002 |
| Zn | 1.01(0.93-1.10) | 0.770 | 0.61(0.45-0.84) | 0.002 |
| Cu | 1.03(0.68-1.56) | 0.875 | 0.12(0.03-0.57) | 0.008 |
| 3,000-10,000 ¥/month(n=633) |  |  |  |  |
| Ca | 0.83(0.78-0.88) | <0.001 | 0.63(0.56-0.71) | <0.001 |
| Mg | 0.76(0.68-0.86) | <0.001 | 0.23(0.16-0.34) | <0.001 |
| Fe | 0.72(0.59-0.90) | 0.003 | 0.09(0.04-0.18) | <0.001 |
| Zn | 0.96(0.93-1.00) | 0.060 | 0.72(0.65-0.81) | <0.001 |
| Cu | 0.80(0.67-0.95) | 0.013 | 0.32(0.21-0.48) | <0.001 |
| >10,000 ¥/month(n=238) |  |  |  |  |
| Ca | 0.87(0.80-0.95) | 0.002 | 0.87(0.75-1.01) | 0.075 |
| Mg | 0.70(0.57-0.86) | 0.001 | 0.28(0.15-0.50) | <0.001 |
| Fe | 0.48(0.32-0.73) | 0.001 | 0.01(0.002-0.06) | <0.001 |
| Zn | 0.91(0.84-0.97) | 0.006 | 0.70(0.58-0.84) | <0.001 |
| Cu | 0.60(0.43-0.86) | 0.005 | 0.28(0.12-0.70) | 0.006 |
| Smoking status |  |  |  |  |
| Never smoking(n=735) |  |  |  |  |
| Ca | 0.85(0.81-0.90) | <0.001 | 0.77(0.70-0.85) | <0.001 |
| Mg | 0.76(0.68-0.85) | <0.001 | 0.31(0.23-0.42) | <0.001 |
| Fe | 0.63(0.51-0.78) | <0.001 | 0.08(0.04-0.16) | <0.001 |
| Zn | 0.93(0.90-0.97) | 0.001 | 0.72(0.65-0.80) | <0.001 |
| Cu | 0.76(0.64-0.90) | 0.002 | 0.36(0.24-0.54) | <0.001 |
| Smoking(n=277)^e^ |  |  |  |  |
| Ca | 0.82(0.76-0.90) | <0.001 | 0.42(0.33-0.54) | <0.001 |
| Mg | 0.78(0.66-0.92) | 0.002 | 0.10(0.05-0.20) | <0.001 |
| Fe | 0.74(0.55-0.98) | 0.038 | 0.02(0.01-0.06) | <0.001 |
| Zn | 0.98(0.93-1.03) | 0.353 | 0.65(0.55-0.76) | <0.001 |
| Cu | 0.77(0.59-1.01) | 0.054 | 0.16(0.07-0.34) | <0.001 |
| History of allergies |  |  |  |  |
| Yes(n=113) |  |  |  |  |
| Ca | 0.94(0.85-1.05) | 0.270 | 0.71(0.56-0.90) | 0.005 |
| Mg | 0.87(0.68-1.10) | 0.246 | 0.15(0.06-0.39) | <0.001 |
| Fe | 0.93(0.60-1.43) | 0.733 | 0.01(0.001-0.14) | <0.001 |
| Zn | 1.02(0.94-1.10) | 0.616 | 0.71(0.54-0.92) | 0.011 |
| Cu | 1.06(0.74-1.52) | 0.749 | 0.32(0.10-0.99) | 0.048 |
| No(n=899) |  |  |  |  |
| Ca | 0.83(0.79-0.87) | <0.001 | 0.68(0.62-0.75) | <0.001 |
| Mg | 0.76(0.69-0.83) | <0.001 | 0.27(0.20-0.36) | <0.001 |
| Fe | 0.65(0.54-0.78) | <0.001 | 0.06(0.03-0.11) | <0.001 |
| Zn | 0.95(0.92-0.98) | 0.001 | 0.70(0.64-0.77) | <0.001 |
| Cu | 0.73(0.62-0.85) | <0.001 | 0.30(0.21-0.44) | <0.001 |
| Family history of cancer |  |  |  |  |
| Yes(n=259) |  |  |  |  |
| Ca | 0.88(0.81-0.95) | 0.001 | 0.72(0.61-0.85) | <0.001 |
| Mg | 0.82(0.70-0.97) | 0.018 | 0.17(0.09-0.33) | <0.001 |
| Fe | 0.74(0.54-1.02) | 0.062 | 0.05(0.01-0.18) | <0.001 |
| Zn | 0.95(0.90-1.00) | 0.063 | 0.66(0.56-0.79) | <0.001 |
| Cu | 0.79(0.60-1.04) | 0.089 | 0.11(0.04-0.28) | <0.001 |
| No(n=753) |  |  |  |  |
| Ca | 0.83(0.78-0.87) | <0.001 | 0.65(0.59-0.72) | <0.001 |
| Mg | 0.75(0.67-0.83) | <0.001 | 0.26(0.19-0.35) | <0.001 |
| Fe | 0.65(0.53-0.79) | <0.001 | 0.06(0.03-0.11) | <0.001 |
| Zn | 0.95(0.92-0.99) | 0.009 | 0.71(0.65-0.79) | <0.001 |
| Cu | 0.75(0.63-0.89) | 0.001 | 0.35(0.24-0.51) | <0.001 |
| Physical Activity |  |  |  |  |
| Low(n=301) |  |  |  |  |
| Ca | 0.98(0.89-1.06) | 0.576 | 0.78(0.66-0.93) | 0.006 |
| Mg | 0.97(0.81-1.16) | 0.761 | 0.38(0.23-0.64) | <0.001 |
| Fe | 1.11(0.81-1.52) | 0.516 | 0.34(0.13-0.84) | 0.020 |
| Zn | 1.04(0.98-1.11) | 0.235 | 0.80(0.67-0.95) | 0.009 |
| Cu | 0.95(0.70-1.29) | 0.729 | 0.15(0.06-0.37) | <0.001 |
| Moderate (n=393) |  |  |  |  |
| Ca | 0.84(0.78-0.90) | <0.001 | 0.69(0.60-0.79) | <0.001 |
| Mg | 0.79(0.69-0.91) | 0.001 | 0.25(0.16-0.38) | <0.001 |
| Fe | 0.65(0.50-0.85) | 0.002 | 0.03(0.01-0.08) | <0.001 |
| Zn | 0.95(0.91-1.00) | 0.044 | 0.73(0.64-0.83) | <0.001 |
| Cu | 0.81(0.65-1.01) | 0.063 | 0.36(0.22-0.60) | <0.001 |
| Violent (n=318) |  |  |  |  |
| Ca | 0.74(0.67-0.82) | <0.001 | 0.65(0.55-0.77) | <0.001 |
| Mg | 0.52(0.42-0.64) | <0.001 | 0.16(0.09-0.28) | <0.001 |
| Fe | 0.36(0.25-0.54) | <0.001 | 0.01(0.003-0.05) | <0.001 |
| Zn | 0.86(0.80-0.91) | <0.001 | 0.61(0.52-0.73) | <0.001 |
| Cu | 0.55(0.41-0.74) | <0.001 | 0.35(0.19-0.64) | 0.001 |

a. Unconditional logistic regression model was used for subgroup analysis.

b. Model 1: Unadjusted model.

c. Model 2: Adjusted covariates in model 2 (except for corresponding hierarchical variables).

d. Middle school and below included primary school and below and middle school.

e. Smoking included former smoking, and current smoking.


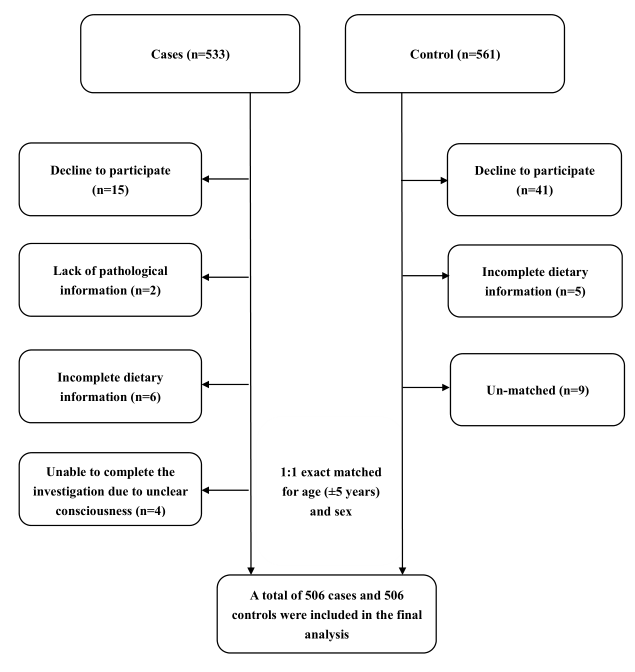


Figure S1. The flow chart of the study population.
